# Copper Alloy Design for Preventing Sulfur-Induced Embrittlement in Copper

**DOI:** 10.3390/ma17020350

**Published:** 2024-01-10

**Authors:** Minkyu Ahn, Jinwoo Park, Gyeongsik Yu, Sangeun Kim, Dong-Keun Cho, Hyung-Ha Jin, Chansun Shin

**Affiliations:** 1 Department of Materials Science and Engineering, Myongji University, 116 Myongji-ro, Cheoin-gu, Yongin 17058, Republic of Korea; 2 Korea Atomic Energy Research Institute, 111, Daedeok-daero 989, Yuseong-gu, Daejeon 34057, Republic of Korea

**Keywords:** sulfur-induced embrittlement, copper alloys, vacancy binding

## Abstract

This study presents an experimental approach to address sulfur-induced embrittlement in copper alloys. Building on recent theoretical insights, we identified specific solute elements, such as silicon and silver, known for their strong binding affinity with vacancies. Through experimental validation, we demonstrated the effectiveness of Si and Ag in preventing sulfur-induced embrittlement in copper, even though they are not typical sulfide formers such as zirconium. Additionally, our findings highlight the advantages of these elements over traditional solutes, such as their high solubility and propensity to accumulate along grain boundaries. This approach may have the potential to be applied to other metals prone to sulfur-induced embrittlement, including nickel, iron, and cobalt, offering broader implications for materials engineering strategies and alloy development.

## 1. Introduction

Sulfur, when present in copper and copper-based alloys, typically induces intergranular embrittlement, leading to a significant reduction in ductility [1,2,3,4,5,6]. This detrimental effect on ductility exhibits distinct temperature-dependent behavior, with a sharp decrease observed around 300 °C, a minimum between roughly 450 and 600 °C, and a gradual increase at temperatures exceeding 600 °C. This temperature-driven phenomenon is commonly referred to as intermediate temperature embrittlement (ITE), which is primarily attributed to intergranular failure [7]. The occurrence of ITE in copper and its alloys bears substantial implications in industrial applications. ITE may restrict the utilization of certain deformation processes, often necessitating either room-temperature or high-temperature deformation processing. Moreover, ITE plays a pivotal role in assessing the elevated temperature performance and long-term service viability of copper and its alloys in sulfur-containing environments [8,9], thereby influencing their selection for engineering applications.

To mitigate the detrimental effects of sulfur-induced embrittlement, traditional approaches have involved the addition of specific solutes, with the effectiveness of certain elements confirmed by trial and error. For instance, minute quantities of yttrium in a copper–zinc alloy enhance ductility at intermediate temperatures [10], while the inclusion of zirconium in a copper–chromium alloy suppresses dynamic embrittlement by sulfur at 200 °C [11]. Similarly, small additions of elements like yttrium, cerium, lanthanum, calcium, and boron have been shown to improve the ductility of pure copper and copper–tin alloys with poor hot ductility [12,13]. Furthermore, the introduction of elements such as titanium, zirconium, or vanadium to sulfur-containing copper alloys can reverse the retardation of recrystallization caused by sulfur [14].

Despite these advances, the selection of alloying elements has primarily relied on trial and error, as the mechanisms through which sulfur reduces the mechanical properties of copper and specific solutes’ roles in mitigating these effects remain incompletely understood. One suggested mechanism for the adverse effects on mechanical properties involves a reduction in grain boundary cohesion due to the segregation of sulfur to these boundaries within copper [15]. Another mechanism entails the formation of sulfide particles along grain boundaries, leading to intergranular fracture. Recent research has unveiled stable micro- and nanoscale Cu_2_S precipitates primarily forming along grain boundaries in sulfur-containing ultrahigh-purity copper, and these precipitates act as nucleation sites for small cavities that initiate cracks [16].

In accordance with these mechanisms, the ability of solutes to mitigate adverse effects is often attributed to their ability to scavenge grain boundary sulfur, thus strengthening grain boundaries by reducing sulfur segregation. Strengthening of grain boundaries may also occur through the segregation of elements along them, inhibiting sulfur segregation by repelling sulfur from the grain boundaries. Additionally, certain elements may form metallic sulfides, preventing the formation of copper sulfides along grain boundaries. The precise effects exerted by individual alloying elements, however, still remain unclear. 

Recent theoretical and numerical investigation has elucidated key aspects of sulfur in copper and its alloys [17]. One study has demonstrated that the solubility of sulfur in copper is inherently limited, prompting sulfur to form stable sulfur–vacancy complexes that exhibit rapid diffusion toward and through grain boundaries or dislocations. The high mobility of sulfur–vacancy defect pairs subsequently leads to the rapid formation of Cu-S compounds at segregation sites, notably along grain boundaries. In summary, sulfur combines with vacancies, leading to the rapid formation of Cu_2_S along grain boundaries. These particles serve as initiators for cracks, which then propagate along the grain boundaries, ultimately resulting in intergranular cracking.

Based on the insights from these investigations, it is conceivable that alloying elements exhibiting attractive interactions with vacancies or the ability to form more stable sulfur compounds than Cu-S compounds may enhance the mechanical properties of copper alloys by competing with sulfur for grain boundary segregation. Moreover, recent advances in computational materials science facilitate the identification of such elements. For example, the binding energy of elements with vacancies in copper can be accurately computed and analyzed using first-principles calculations based on density functional theory (DFT) [18]. The formation energies of sulfides can be accessed within the Open Quantum Materials Database (OQMD) [19], which provides comprehensive datasets encompassing DFT-calculated and experimentally measured formation energies of sulfides and other compounds. 

These computational tools collectively offer opportunities to enhance our understanding of the effects of previously reported ‘de-embrittling’ solutes and systematically identify such elements in copper alloys. If successful, this approach may extend to other 3-D transition metals such as iron [20,21], cobalt [22], and nickel [23,24], where sulfur embrittlement phenomena are prevalent. It is worth noting that sulfur also diffuses rapidly due to the formation of sulfur–vacancy pairs in these 3-D transition metals [25]. 

In this study, we employ the previously discussed computational tools to identify new elements capable of mitigating sulfur-induced embrittlement in copper. Subsequently, we validate the effectiveness of these elements through experimental investigations involving the fabrication of copper alloys. The paper is structured as follows: we begin with the experimental procedures, followed by a detailed analysis of the selection of alloying elements in the Results and Discussion section, encompassing a discussion of our experimental findings. The paper concludes with a summary and outlines directions for future research studies.

## 2. Materials and Methods

**Figure 1:**
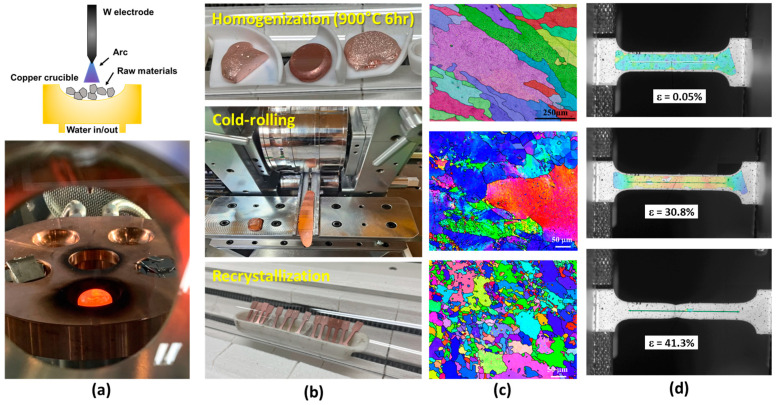
(**a**) Vacuum arc melting and ingot casting process; (**b**) homogenization, cold rolling, and recrystallization process; (**c**) electron backscatter diffraction mapping of the ingot, cold-rolled plate, and fully recrystallized Cu; and (**d**) digital image correlation during tensile testing.

Copper binary alloys, comprising 0.5 atomic percent (at%) Si, Ag, and Zr, were prepared using a vacuum arc melting furnace (Samhan Vacuum Development Co., Ltd., Paju, Republic of Korea). The rationale for selecting these specific alloying elements will be elucidated in the subsequent section. Each alloy was cast into ingots weighing 10 g apiece. To assess the influence of sulfur incorporation, 0.2 at% S was introduced into each copper binary alloy. The composition of each alloy can be found in Table 1. Figure 1a provides schematics depicting the vacuum arc melting process, along with an actual image of the molten ingot. To ensure the thorough mixing of raw materials during the melting process, solidified ingots were inverted and subsequently re-melted within the copper crucible at least five times or more.

The manufactured ingots underwent a heat treatment process at 900 °C for 6 h within a tube furnace under an argon atmosphere. This homogenization treatment was employed to eliminate solute segregation within the ingots. Subsequently, cold rolling was carried out using a rolling mill, resulting in a reduction in the ingots to an average thickness of 1 mm, corresponding to approximately 80% plastic deformation, as illustrated in Figure 1b. The cold-rolled plates were subjected to isothermal annealing at temperatures of 200 °C, 400 °C, and 600 °C, each for a duration of 30 min. The hardness of the annealed alloys was measured employing a Vickers indenter with a 0.3 kgf load. Notably, annealing at 600 °C for 30 min led to fully recrystallized microstructures. Figure 1c presents electron backscatter diffraction mapping of the ingot, cold-rolled plate, and fully recrystallized specimen of pure copper (Cu), presented from top to bottom. Consequently, tensile specimens were further annealed at 600 °C for 30 min to facilitate additional analysis.

The tensile specimen had the following dimensions: a total length of 26 mm, a gage length of 9 mm, a width of 2 mm, and a thickness of 1 mm. Tensile testing was conducted at room temperature using a ZwickRoell Z005 tensile testing machine (ZwickRoell, Ulm, Germany) and employing a strain rate of 10^−3^/s. To capture and measure strain during the tensile test, digital image correlation (DIC) was employed. The GOM Correlate program facilitated this strain measurement process. Figure 1d illustrates an image of the DIC experiment conducted for strain analysis.

The examination of the samples’ microstructure was conducted through scanning electron microscopy (SEM). To measure the distribution of solute atoms, energy-dispersive X-ray spectroscopy (EDS) was employed. For SEM analysis, the samples underwent a preparation process involving mechanical polishing using SiC papers and diamond suspensions (with particle sizes of 3 and 0.5 µm). Following this polishing step, the surface was etched utilizing a mixed solution comprising nitric acid (HNO_3_, 50%) and distilled water (50%).

## 3. Results

**Figure 2:**
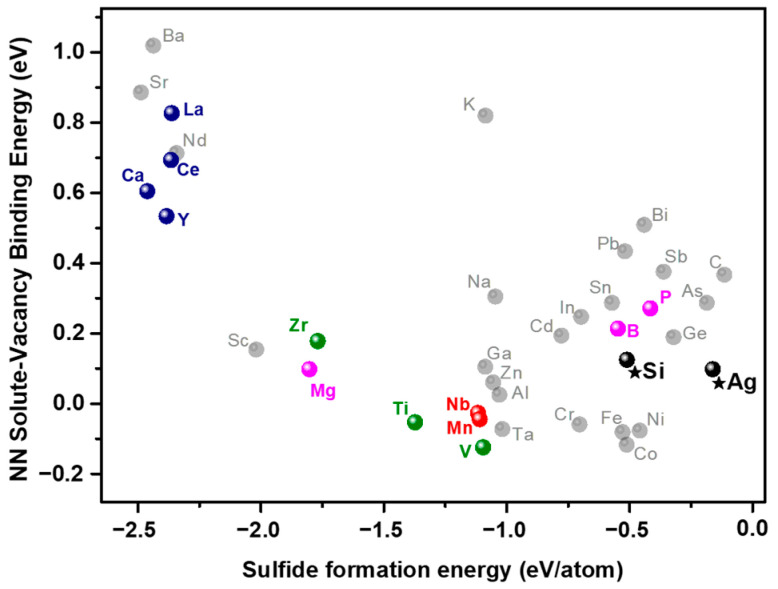
Solute–vacancy binding energy vs. sulfide formation energy diagram for 37 alloying elements.

The nearest-neighbor (NN) solute–vacancy binding energies in Cu, as referenced in [18], along with sulfide (MS) formation energies retrieved from www.oqmd.org/materials/composition (accessed on 7 December 2023) [19], for a total of 37 alloying elements, are graphically represented in Figure 2. Whenever experimental sulfide formation energy values were available, they were included in the plot. In cases where experimental values were unavailable, DFT-calculated formation energies were utilized.

Elements exhibiting positive solute–vacancy binding energies indicate favorable binding with neighboring vacancies in Cu. For instance, elements like Ba, Sr, and La display significantly large positive vacancy binding energies. In contrast, elements such as Ti, Nb, V, and Ta exhibit negative solute–vacancy binding energies, suggesting a repulsive interaction with vacancies. Consequently, Ba, Sr, La, and similar elements are expected to have a stronger affinity for neighboring vacancies, while Ti, Nb, V, and Ta are predicted to repel vacancies. Furthermore, the sulfide formation energies for elements like Ba, Sr, and La exhibit notably large negative values, approximately −2.3 eV/atom. In comparison, the formation energies of CuS and Cu_2_S are considerably lower at −0.285 and −0.275 eV/atom, respectively. Consequently, sulfides such as BaS, LaS, and CeS demonstrate higher stability when compared to CuS or Cu_2_S. It is worth noting that formation energy can serve as a scale for thermodynamic stability, as the change in Gibbs free energy due to temperature is typically on the order of a few tens of millielectronvolts (meV) at room temperature, while the energy associated with chemical reactions ranges from hundreds of meV to several electronvolts (eV). It is important to emphasize that the vacancy binding energies are calculated for elements as solutes in Cu. On the other hand, the sulfide formation energies of each element do not consider the presence of solute elements in Cu. Consequently, the propensity of elements to form sulfides in Cu can only be comparatively assessed at best.

As depicted in Figure 2, it is noteworthy that elements such as La, Ce, Ca, and Y (indicated by the navy color), which have been previously identified as effective de-embrittlers in Cu [12] and Cu-4.4 mol% Sn alloy [13], cluster together with both high negative values for sulfide formation energy and a notably high positive value for solute–vacancy binding energy. The aforementioned study reported the detection of trace sulfur impurities at the intergranular fracture surface of the alloy in the absence of added elements. In contrast, alloys containing Y exhibited the presence of dispersed sulfide particles containing yttrium. Each of these elements is considered a strong sulfide former, contributing to a reduction in sulfur segregation at grain boundaries due to sulfide formation [13]. 

The addition of small quantities of Ti, Zr, or V into sulfur-containing Cu specimens has been reported to result in the formation of finely dispersed sulfide particles of these additive elements within the cast structures [14]. Notably, the sulfide formation energies of these elements (indicated by the green color in Figure 2) are lower than those of CuS or Cu_2_S, as depicted. Additionally, the incorporation of Nb or Mn (highlighted in red) has been documented to mitigate embrittlement in copper alloys [7]. While the precise mechanism behind this reduced embrittlement has not been reported, it can be inferred that sulfides formed by the additive elements may play a role in mitigating the adverse effects of sulfur.

Alloying with 0.1 mol% of B, Mg, or P (indicated in magenta in Figure 2) has shown a substantial improvement in the poor ductility of Cu-8 mass% Sn at intermediate temperatures [26]. While this study did not establish a direct correlation between the enhanced ductility and the formation of sulfide particles or the reduction in sulfur’s effects on grain boundaries, the effects of Mg could potentially be attributed to the formation of Mg sulfide particles. On the other hand, the effects of the addition of B or P cannot be solely explained by the formation of sulfide particles. These elements, however, possess positive vacancy binding energies, suggesting that they favorably bind with neighboring vacancies. Consequently, these solute atoms are expected to impede the formation of sulfur–vacancy complexes and thereby hinder or prevent the formation of copper sulfides at grain boundaries.

**Figure 3:**
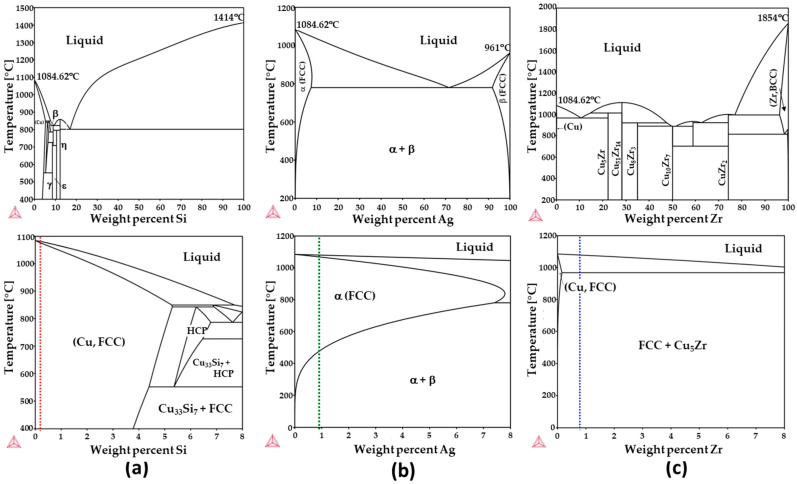
Phase diagrams of (**a**) Cu–Si, (**b**) Cu–Ag, (**c**) and Cu–Zr with compositions ranging from 0 to 100 wt% and 0 to 8 wt%. Dashed lines in the bottom diagrams depict the composition employed in this study.

Based on a comparative analysis with results reported in references [7,13,14,26], it becomes evident that elements exhibiting either a pronounced tendency for sulfide formation or a strong affinity for vacancy binding can effectively reduce sulfide-induced embrittlement. To further investigate and validate this hypothesis, we selected Si and Ag as exemplary elements. Both Si and Ag possess positive solute–vacancy binding energies while exhibiting a relatively lower tendency for sulfide formation. Moreover, Si exhibits a noteworthy solubility in Cu, approximately around 8 at%, forming a single solid solution phase within this range, as illustrated in Figure 3a. In contrast, the Cu-Ag phase diagram in Figure 3b demonstrates the formation of Ag-rich precipitates within the Cu matrix under equilibrium conditions. Although the magnitude of both vacancy binding energy and sulfide formation energy for Si and Ag may be somewhat lower compared to values observed for other elements, their higher solubility can be considered an advantageous feature. It is worth noting that there exists a moderate correlation between solute–vacancy binding energy and the size of the solute atoms [18], and the solubility of larger solutes with significant vacancy binding energy tends to be limited. In addition, Zr was also investigated for comparative purposes. The phase diagram in Figure 3c reveals that the maximum solid solubility of Zr in Cu is approximately 0.12 at% at 972 °C, with the formation of an intermetallic phase (Cu_5_Zr) occurring at room temperature.

**Figure 4:**
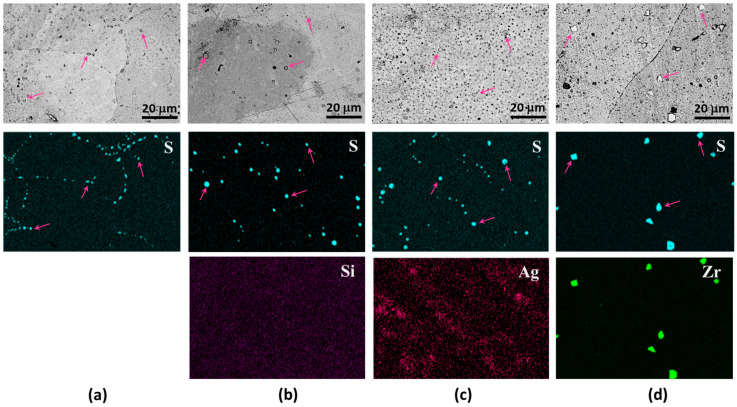
SEM micrographs and EDS maps of as-cast Cu alloys with sulfur addition: (**a**) Cu-0.2 at% S, (**b**) Cu-0.5 at% Si-0.2 at% S, (**c**) Cu-0.5 at% Ag-0.2 at% S, and (**d**) Cu-0.5 at% Zr-0.2 at% S.

Figure 4a presents an SEM micrograph of Cu-0.2 at% S, accompanied by an EDS mapping image highlighting sulfur distribution. Within these micrographs, spherical and elliptical particles, with some being indicated by arrows for clarity, are discernible along grain boundaries. The EDS map corroborates that these particles exhibit a higher sulfur concentration, thus confirming their identity as copper sulfide particles. Turning to the microstructures of Cu-0.5 at% Si-0.2 at% S, depicted in Figure 4b, the distribution of sulfide particles deviates from the alignment along the grain boundaries, now being randomly scattered within the grains. The EDS map for Si reveals no discernible Si-rich phase and no spatial correlation with sulfur. In Figure 4c, the SEM image and EDS map for S in Cu-0.5 at% Ag-0.2 at% S reveal the formation of sulfide particles within the grains. However, the EDS map for Ag demonstrates an uneven distribution of Ag atoms, with sulfide particles exhibiting no clear correlation with Ag-rich regions. Figure 4d illustrates SEM and EDS results for Cu-0.5 at% Zr-0.2 at% S. Similar to the other Cu alloys, sulfide particles are randomly dispersed within the grains rather than aligned along grain boundaries. The EDS map for Zr distinctly identifies the sulfide particles as zirconium sulfide, as anticipated from the negative formation enthalpy values presented in Figure 2.

**Figure 5:**
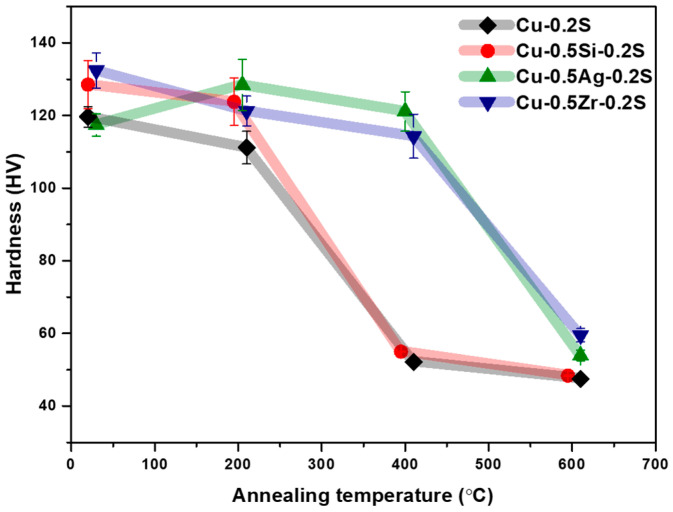
Vickers hardness measured on homogenized and cold-rolled specimens after annealing for 30 min at the temperatures indicated.

The hardness results for the annealed alloys are presented in Figure 5. Notably, the hardness values exhibited a significant decline within a specific temperature range, corresponding to the occurrence of recrystallization. Earlier research findings indicated that recrystallization typically takes place between 300 and 400 °C for Cu–Si alloys, 400 and 500 °C for Cu–Ag alloys, and 500 and 600 °C for Cu–Zr alloys [27]. As demonstrated in Figure 5, both Cu-Ag and Cu-Zr alloys exhibit a suppressed tendency for recrystallization, attributed to the formation of precipitates during the recrystallization process. A prior study elucidated that the addition of Zr to Cu effectively postpones the initiation of recrystallization to higher temperatures. This delay is attributed to the presence of intermetallic Cu_5_Zr precipitates within the alloys, which inhibit grain boundary movement [28,29].

**Figure 6:**
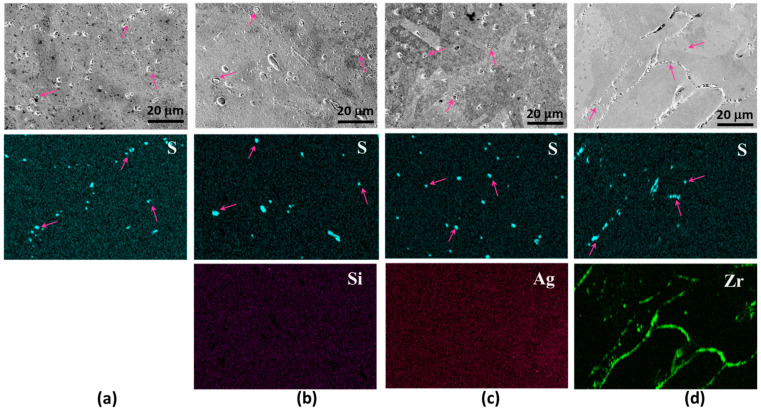
SEM micrographs and EDS maps of homogenized, cold-rolled, and annealed Cu alloys with sulfur addition: (**a**) Cu-0.2 at% S, (**b**) Cu-0.5 at% Si-0.2 at% S, (**c**) Cu-0.5 at% Ag-0.2 at% S, (**d**) Cu-0.5at%Zr-0.2at%S.

The presence and distribution of sulfide particles were observed in samples following processes of homogenization, cold rolling, and annealing. The microstructures of the cold-rolled and annealed samples are illustrated in Figure 6. Similar to the as-cast alloys, sulfides were observed along grain boundaries in the Cu-0.2 at% S specimen. In contrast, Cu-0.5 at% Si-0.2 at% S and Cu-0.5 at% Ag-0.2 at% S samples displayed sulfides formed within the grains. In the recrystallized specimen of Cu-0.5 at% Ag-0.2 at% S, Ag exhibited a more uniform distribution compared to the as-cast specimen, with no evident Ag precipitates detected. The Cu-0.5 at% Zr-0.2 at% S alloy sample revealed the formation of Zr intermetallics along grain boundaries. As discussed earlier, the inclusion of Zr as an alloying element demonstrated its effectiveness in inhibiting grain size increase through the formation of intermetallic Cu_5_Zr precipitates.

**Figure 7:**
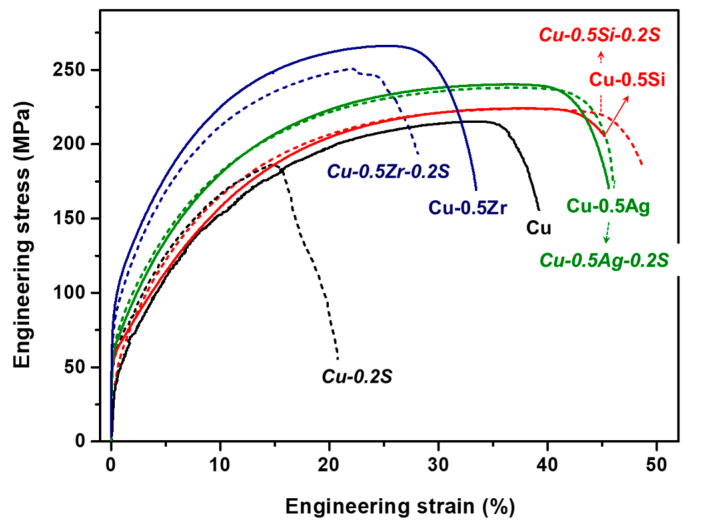
Engineering stress–strain curves of pure copper and copper alloys. Dashed lines represent specimens with sulfur addition.

The mechanical properties of the alloys, in addition to pure copper, are elucidated through the stress–strain (SS) curves obtained from tensile tests, as depicted in Figure 7. The ultimate tensile strength is observed to be highest for Cu-0.5 at% Zr (~265 MPa), followed by Cu-0.5 at% Ag (~240 MPa), Cu-0.5 at% Si (224 MPa), and Cu (215 MPa). Note that the tensile strengths of each alloy follow the same order as the Vickers hardness results at a 600 °C annealing temperature presented in Figure 5. The ductility of the Cu, Cu-Ag, and Cu-Si samples exceeds 40%, except for the Cu-Zr alloy. Tensile curves for samples containing sulfur are represented by dashed curves. Notably, the ductility of pure copper experiences a significant decline when sulfur is introduced. In particular, the Cu-0.2 at% S alloy exhibited a brittle fracture. Furthermore, the Cu-0.5 at% Zr alloy also exhibits reduced elongation when sulfur is added, although to a lesser extent than pure copper. This decrease in elongation is attributed to the formation of intermetallic phases along grain boundaries, as evidenced in Figure 6d. The Cu-0.5 at% Zr alloy exhibited the formation of finer intermetallic phases [27]. The reason behind the formation of Zr intermetallic phases along grain boundaries in the presence of sulfur remains unclear and requires further investigation. Previous studies [11,30] have shown that the addition of zirconium to a Cu-Cr alloy improved ductility by forming zirconium sulfide and reducing the amount of impurity sulfur segregated at grain boundaries (sulfur scavenging). However, it is important to note that the Zr addition in those studies was of a significantly smaller amount (0.04~0.05 wt%) compared to that considered in the current study, approximately 0.71 wt% Zr. On the other hand, both the 0.5 Ag and 0.5 Si alloys exhibit negligible decreases in elongation, attributed to alterations in sulfide particle distribution. Further analysis involves the examination of fracture surfaces from tensile specimens of pure copper, Cu-0.2 at% S, and Cu-0.5 at% Si-0.2 at% S alloy.

**Figure 8:**
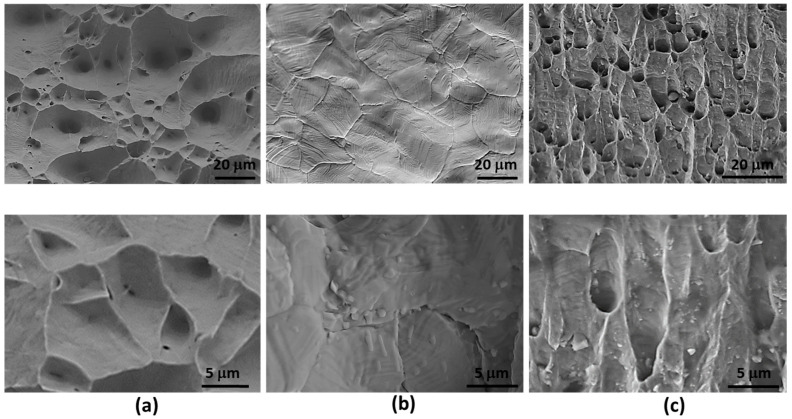
SEM micrographs of fractured surface of tensile specimens of (**a**) pure copper, (**b**) Cu-0.2 at% S, and (**c**) Cu-0.5 at% Si-0.2 at% S.

Figure 8 displays SEM images depicting fracture surfaces from tensile specimens of pure copper, Cu-0.2 at% S alloy, and Cu-0.5 at% Si-0.2 at% S alloy. A transgranular fracture surface is evident in both the pure copper (a) and Cu-0.5 at% Si-0.2 at% S (c) specimens, while an intergranular fracture surface is observed in the Cu-0.2 at% S alloy (b). In Figure 8a,c, spherical dimples are visible, corresponding to micro-cavities that initiate crack formation. Notably, the dimple size is smaller for the Cu-0.5 at% Si-0.2 at% S specimen, as these dimples form around sulfide particles within grains. Considerable plastic deformation occurs prior to fracture around these dimples. The intergranular facets on the fracture surface of the Cu-0.2 at% S specimen exhibit striations (Figure 8b). These steps on the intergranular facets are a result of segregation, serving to minimize grain boundary energy [31]. Some of these intergranular facets indicate the presence of fine intergranular particles, with sulfur peaks being detected in EDS analysis.

**Figure 9:**
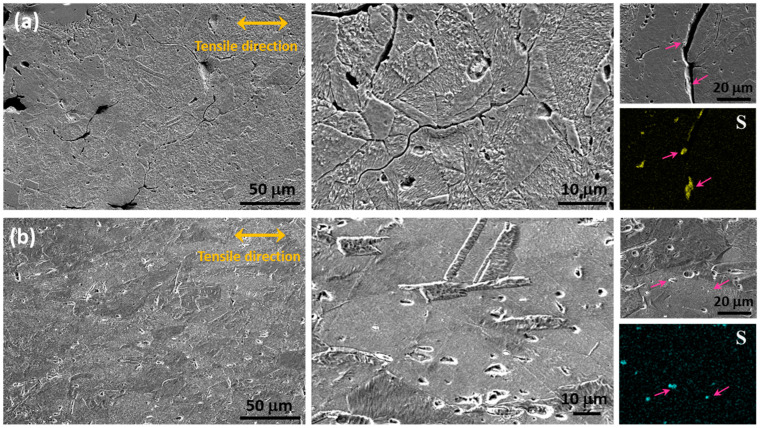
SEM micrographs in cross-section close to the tensile fracture surface: (**a**) Cu-0.2 at% S and (**b**) Cu-0.5 at% Si-0.2 at% S.

SEM micrographs of the cross-section near the fracture surface of the Cu-0.2 at% S and Cu-0.5 at% Si-0.2 at% S samples subjected to tension testing are presented in Figure 9. The directions of tensile loading are indicated by arrows. In Figure 9a, the Cu-0.2 at% S specimen reveals the presence of grain boundary microcracking near the fracture site while largely preserving the initial grain morphology. Extensive intergranular damage accumulation is evident within the material. EDS analysis was specifically conducted on a cross-section, focusing on the areas surrounding the grain boundary cracks in the Cu-0.2 at% S specimen. This analysis detects sulfur within the intergranular fracture cracks, pinpointing sulfide particles at the grain boundary as the initiation point for cracking. These micrographs provide clear evidence that sulfur particles formed at grain boundaries contribute to grain boundary cracking and embrittlement. In contrast, the Cu-0.5 at% Si-0.2 at% S alloy displayed a notably different behavior, as shown in Figure 9b. Grain boundary cracking is scarcely observed in this specimen. Instead, the grains elongate in the tensile direction. Sulfur was detected within the sulfide particles of the Cu-0.5 at% Si-0.2 at% S specimen, which are distributed inside the grains. In specimens containing 0.5 at% silicon, dispersed particles become apparent, and no grain boundary cracking is observed. Hence, it can be concluded that the enhancement in ductility attributed to the inclusion of silicon and silver stems from the prevention of copper sulfide formation along grain boundaries. Silicon, although not a strong sulfide former, has the capability to form copper sulfides within grains while inhibiting their formation along grain boundaries by binding with vacancies and thus impeding the creation of rapidly diffusing sulfur–vacancy complexes. However, it is important to note that this observation does not directly confirm the presence of Si–vacancy complexes, for which techniques such as PALS (Positron Annihilation Lifetime Spectroscopy) will be conducted in future studies.

**Figure 10:**
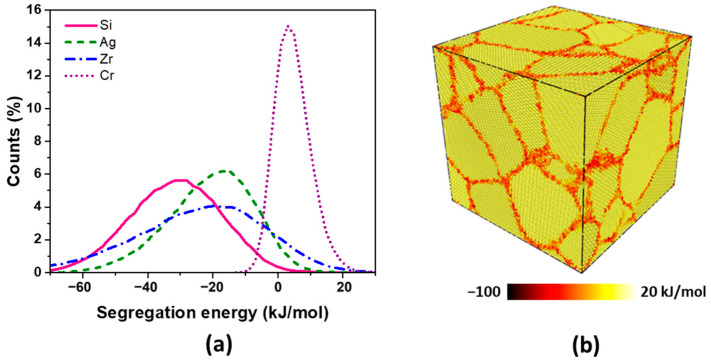
(**a**) Grain boundary segregation energy spectra for Si, Ag, Zr, and Cr in Cu; (**b**) Si solute segregation energies in a Cu polycrystal (20 × 20 × 20 nm^3^ atomistic simulation volume with 16 randomly oriented grains).

Furthermore, in addition to their higher alloying content and their inclination to avoid forming intermetallic phases, Si and Ag atoms also exhibit a tendency to segregate around grain boundaries. Recently, a machine learning framework has been developed to accurately predict the relaxed segregation energy of solute atoms at grain boundary sites [32]. Figure 10b provides an illustration of the segregation energy of Si, calculated as the relaxed energy difference between a solute Si atom occupying a grain boundary site and a bulk site within a 20 × 20 × 20 nm^3^ atomistic simulation of a Cu polycrystal volume containing 16 randomly oriented grains. A negative energy difference, referred to as segregation energy, signifies the occurrence of solute segregation around grain boundaries. The grain boundary segregation energy spectra for Si, Ag, Zr, and Cr in Cu are presented in Figure 10a. Among these elements, Si, Ag, and Zr exhibit a proclivity to segregate around copper grain boundaries. This tendency can be advantageous in scenarios where sulfur diffusion from the external environment into copper primarily occurs through these grain boundaries. Solutes with a preference for segregating around grain boundaries in Cu serve as effective anchors for sulfur diffusion and the formation of Cu_2_S particles. Consequently, the migration of sulfur from the external environment may be obstructed by solute atoms, warranting future research to explore this phenomenon. This investigation should encompass the validation of grain boundary segregation of solute elements through the utilization of atom probe tomography.

## 4. Conclusions

Through an examination of solute–vacancy binding energy and sulfide formation energy (depicted in Figure 2), we observed that previously identified ‘de-embrittling’ solute elements in the literature exhibit strong tendencies to form sulfides or bind with neighboring vacancies. We propose that alloying elements showing one or both of these tendencies can impede sulfur migration toward grain boundaries, thereby inhibiting grain boundary sulfide formation and embrittlement. To verify this hypothesis, we selected new alloying elements—Si and Ag—along with Zr, a known ‘de-embrittling’ solute element, and manufactured binary copper alloys with added sulfur. These alloys were then subjected to mechanical tests and microstructural analysis.

Both Si and Ag were found to hinder the formation of grain boundary sulfides. Sulfide particles were observed within the grains, and tensile testing revealed that these elements effectively prevented sulfur-induced embrittlement and intergranular fracture. Our findings suggest that elements that do not strongly form sulfides but exhibit a strong binding affinity with vacancies can be effective in reducing sulfur embrittlement. These elements offer advantages over sulfide-forming solutes like Zr, which often have limited solubility and tend to form intermetallic compounds. Additionally, these elements preferentially accumulate along grain boundaries, serving as a barrier to incoming sulfur from external sources.

While our study provides compelling evidence, additional research is necessary to directly confirm alterations in vacancy-related defects using Positron Annihilation Lifetime Spectroscopy (PALS) and to investigate the grain boundary segregation of solutes using atom probe tomography (ATP). The methodology outlined in this study can be applied to mitigate sulfur-induced embrittlement in other metals, such as Ni, Fe, and Co, which share similar embrittlement mechanisms. Exploring these common mechanisms in diverse metal systems could yield valuable insights and solutions.

## Figures and Tables

**Table 1:** The composition of the alloys used in this study. All the numbers are given in at%.

S (at%)	Cu	Cu-Si Alloy	Cu-Ag Alloy	Cu-Zr Alloy
0	Cu	Cu-0.5Si	Cu-0.5Ag	Cu-0.5Zr
0.2	Cu-0.2S	Cu-0.5Si-0.2S	Cu-0.5Ag-0.2S	Cu-0.5Zr-0.2S

## Data Availability

Data are contained within the article, further inquiries can be directed to the corresponding author.
